# ChatGPT’s performance in German OB/GYN exams – paving the way for AI-enhanced medical education and clinical practice

**DOI:** 10.3389/fmed.2023.1296615

**Published:** 2023-12-13

**Authors:** Maximilian Riedel, Katharina Kaefinger, Antonia Stuehrenberg, Viktoria Ritter, Niklas Amann, Anna Graf, Florian Recker, Evelyn Klein, Marion Kiechle, Fabian Riedel, Bastian Meyer

**Affiliations:** ^1^Department of Gynecology and Obstetrics, Klinikum Rechts der Isar, Technical University Munich (TU), Munich, Germany; ^2^Department of Gynecology and Obstetrics, Friedrich–Alexander-University Erlangen–Nuremberg (FAU), Erlangen, Germany; ^3^Department of Gynecology and Obstetrics, Bonn University Hospital, Bonn, Germany; ^4^Department of Gynecology and Obstetrics, Heidelberg University Hospital, Heidelberg, Germany

**Keywords:** artificial intelligence, ChatGPT, medical education, machine learning, obstetrics and gynecology, students

## Abstract

**Background:**

Chat Generative Pre-Trained Transformer (ChatGPT) is an artificial learning and large language model tool developed by OpenAI in 2022. It utilizes deep learning algorithms to process natural language and generate responses, which renders it suitable for conversational interfaces. ChatGPT’s potential to transform medical education and clinical practice is currently being explored, but its capabilities and limitations in this domain remain incompletely investigated. The present study aimed to assess ChatGPT’s performance in medical knowledge competency for problem assessment in obstetrics and gynecology (OB/GYN).

**Methods:**

Two datasets were established for analysis: questions (1) from OB/GYN course exams at a German university hospital and (2) from the German medical state licensing exams. In order to assess ChatGPT’s performance, questions were entered into the chat interface, and responses were documented. A quantitative analysis compared ChatGPT’s accuracy with that of medical students for different levels of difficulty and types of questions. Additionally, a qualitative analysis assessed the quality of ChatGPT’s responses regarding ease of understanding, conciseness, accuracy, completeness, and relevance. Non-obvious insights generated by ChatGPT were evaluated, and a density index of insights was established in order to quantify the tool’s ability to provide students with relevant and concise medical knowledge.

**Results:**

ChatGPT demonstrated consistent and comparable performance across both datasets. It provided correct responses at a rate comparable with that of medical students, thereby indicating its ability to handle a diverse spectrum of questions ranging from general knowledge to complex clinical case presentations. The tool’s accuracy was partly affected by question difficulty in the medical state exam dataset. Our qualitative assessment revealed that ChatGPT provided mostly accurate, complete, and relevant answers. ChatGPT additionally provided many non-obvious insights, especially in correctly answered questions, which indicates its potential for enhancing autonomous medical learning.

**Conclusion:**

ChatGPT has promise as a supplementary tool in medical education and clinical practice. Its ability to provide accurate and insightful responses showcases its adaptability to complex clinical scenarios. As AI technologies continue to evolve, ChatGPT and similar tools may contribute to more efficient and personalized learning experiences and assistance for health care providers.

## Introduction

Chat Generative Pre-Trained Transformer (ChatGPT) is an artificial learning and large language model tool that was first released by OpenAI on 30 November 2022. Its web-browser-based conversational interface was built atop the large language model Generative Pretrained Transformer 3 (GPT-3), which had first been released in 2020 (1). Reinforcement learning techniques were further utilized to train the model on a dataset of 570 gigabytes of text, which allowed ChatGPT to establish probabilistic relationships between words and to perform natural language processing and generation tasks (2).

The theoretical foundation for the development of ChatGPT was grounded on the idea that language can be learned using patterns and rules found in large text corpora (3). ChatGPT generates its responses using deep learning algorithms that are trained on vast sets of text data, which enables the model to understand the structure, syntax, and semantics of natural language (4, 5). ChatGPT analyzes the input text and generates a response based on the patterns and rules it has learned during the training process (3). The model selects the most probable answer from a large set of potential responses and ranks these responses according to their likelihood of being coherent with and appropriate for the input text so as to mimic human language and provide relevant information or assistance. Prior to ChatGPT, large language models had predominantly been confined to the artificial intelligence (AI) research community. However, such models were not widely adopted by the general public due to their technical complexity and lack of accessibility. However, ChatGPT was different because it introduced a conversational interface that enabled users to interact with the AI in a more human-like manner (6).

The inclusion of ChatGPT or other AI applications in medical education and clinical practice may have the potential to transform the way in which students – as well doctors and patients – acquire knowledge in biomedical sciences (7, 8). In theory, by leveraging their vast knowledge base and real-time information processing capabilities, AI applications can offer personalized learning experiences to medical students. They can adapt to individual learning styles, providing tailored resources and interactive simulations to enhance understanding. Additionally, AI applications could provide instant feedback on students’ performance, identifying areas of weakness and suggesting targeted improvement strategies. This continuous, adaptive learning approach promises to significantly improve the quality and effectiveness of medical education. This development is still at an early stage, and potential applications and benefits remain rather hypothetical due both to the short time for which they have been available and to the lack of impactful studies on the topic. At this early stage of implementation of AI in medical education and clinical practice, our objective was to gain insights into both the capabilities and limitations of ChatGPT in this regard. One significant challenge is the lack of nuanced understanding and contextual judgment that AI applications currently have, which is crucial in medical training. AI systems might struggle to replicate the complex decision-making processes and ethical considerations inherent in clinical practice. Furthermore, issues around data privacy and security are paramount in medical education, where sensitive health information is involved.

We sought to assess the tool’s ability to demonstrate medical knowledge and to evaluate its performance in the context of problem assessment in obstetrics and gynecology (OB/GYN). In order to achieve this goal, we investigated ChatGPT’s performance by analyzing two datasets: first, the OB/GYN course examinations at the Technical University (TU) Munich, and second, the German medical state licensing exam, with a focus on OB/GYN-related questions. We conducted a quantitative and qualitative analysis and compared the results obtained by ChatGPT with those obtained by the students. We hypothesized that at this early stage of its development, ChatGPT should be capable both of processing medical questions and problems well and of providing a high-quality informative output for medical education. Therefore, we aimed to address and discuss three core questions in our study: (i) How well does ChatGPT perform on standardized written OB/GYN exam questions? (ii) Can ChatGPT potentially be applied as a teaching and learning tool in medical education? (iii) What are the potential applications and limitations of ChatGPT for future use in medical education and clinical practice?

## Materials and methods

### Data acquisition and processing

We established two datasets for analyzing and assessing ChatGPT’s ability to understand medical topics and issues in OB/GYN. The first dataset was obtained from the exams in the OB/GYN course during the clinical stage of medical studies at the University Hospital of the Technical University Munich. The corresponding exams were held on 6 February 2023 (with 154 participants), 22 July 2022 (with 125 participants), 7 February 2022 (with 185 participants), 14 July 2021 (with 149 participants), and 1 February 2021 (with 173 participants). The exams are mandatory for passing the OB/GYN course and are usually held in the eighth semester. They test theoretical knowledge across the entire spectrum of OB/GYN that is taught in lectures and small-group seminars during the semester. Topics include general gynecology, prenatal and perinatal medicine, gynecologic oncology, endocrinology, and reproductive medicine. Multiple-choice questions are provided with five answer options each, only one of which is correct. The exams test both the knowledge and understanding of clinical concepts through clinical case presentations. The answer sheets were evaluated anonymously for our study, and the mean rate of correct answers was calculated for each question individually.

The data of the OB/GYN-related state exam questions were obtained from the online teaching and learning software AMBOSS®. This commercial learning platform is particularly well-regarded for its integrated learning system, which includes detailed medical articles, an extensive question bank for exam preparation, and interactive case studies to enhance clinical understanding. One of the standout features of AMBOSS® is its cross-linked articles, which allow users to easily navigate between related topics, making it an efficient tool for both studying and quick reference in clinical practice (9). The state exam (“Zweiter Abschnitt der Ärztlichen Prüfung”) typically consists of just over 300 multiple-choice questions with five answer options each, only one of which is correct. The individual specialties and topic areas are represented differently based on their general clinical relevance. OB/GYN-related questions account for approximately 5% of all questions on the state exam. The questions consist partly of general-knowledge questions as well as of questions in the form of clinical case presentations. The questions are compiled anew biannually for the state exam by the Institute for Medical and Pharmaceutical Exam Questions (IMPP). The exams are uniform across Germany and are conducted over three consecutive days. Students can answer the multiple-choice questions from the state exams of recent years through the online platform AMBOSS®, where they can receive annotated explanations and links to teaching content for each answer option. AMBOSS® also offers the option to indicate the relative difficulty of a question on a scale of 1–5 for personal self-assessment and provides the mean rate of correct answers for each question on the exam. For our dataset, we used the latest 104 questions available on AMBOSS® at the time of data acquisition, and we limited the questions to those related to the field of OB/GYN from the state exams from autumn 2022, spring 2022, autumn 2021, and spring 2021.

We standardized our input formats for both datasets in ChatGPT in line with Gilson et al. (10). This standardization was crucial as it is widely recognized that the phrasing of a prompt can significantly influence the AI’s response or output. Consequently, we excluded questions that contained images because ChatGPT only accepted textual inputs. Additionally, we removed questions with answers presented in table format. The questions were formatted by presenting the question first, followed by the possible multiple-choice answers, each on a new line with a clear numeration of the answer options. A new chat in ChatGPT was opened for each question.

After establishing the two datasets as described above, we investigated ChatGPT’s ability to correctly answer the exam questions. To do so, we entered the corresponding questions with their five answer options into the chat interface of ChatGPT between 10 and 28 February 2023 during several input sessions (not counted). The response time by ChatGPT is immediate as it requires only seconds to process the question and create a cohesive response without the need for corrections. Questions and answers were obtained in the German language. Access was granted through free registration and login via the website https://openai.com/blog/chatgpt. The most recent version of ChatGPT-3 was used at the time of data analysis, and the tool’s response was documented immediately for each individual question. Version 3 of ChatGPT was the first publicly available version capable of comprehensively answering users’ questions formulated in a free and open-ended manner. At the time of data collection for the present study, the most recent text corpora on which ChatGPT had been trained stemmed from September 2021. Therefore, more recent or innovative medical findings after that date may not have been incorporated into the AI’s knowledge base that could have a negative effect on the quality of output. After data acquisition, data analysis followed as described below.

### Data analysis

In order to compare the accuracy of ChatGPT with that of the students from both datasets, we evaluated each answer manually and only considered a response to be correct if it clearly matched one of the five options. During this stage of data analysis, we disregarded any explanations or justifications of the correct answer as well as comments on the incorrect choices. Furthermore, we classified the level of difficulty for each question based on whether it had been answered correctly by more or fewer than the mean number of students, which was 83% of students for the OB/GYN course and 73% of students for the state exam questions. Thus, we established a simple, yet objective dichotomous characterization of a level of difficulty for each question for further statistical analysis. We then examined whether ChatGPT exhibited any significant differences in its rate of correct responses for the group of easy or difficult questions. Furthermore, we examined whether there was a correlation between the numerical difficulty score (from 1 = “very easy” to 5 = “very difficult”) that had been assigned by AMBOSS® for each question on the one hand and ChatGPT’s performance on the other hand.

In order to further investigate ChatGPT’s ability to process information and provide useful medical information, the multiple-choice questions from both the OB/GYN course and the state exam were assigned to the following two groups:

1. General knowledge questions; for example: *“Which statement about cervical cancer is correct?”*
2. Clinical case presentation; for example: *“A 40-year-old gravida I / para 0 presents to the labor ward on the weekend at 34 + 4 weeks of gestation with complaints of having felt unwell for two days. She reports increasing lower limb edema, nausea, and vomiting since yesterday evening, intermittent visual disturbances and tinnitus since a few hours ago, and right upper quadrant abdominal discomfort. Which diagnostic measure is NOT helpful in this situation?”*

We evaluated ChatGPT’s responses to each question based on five variables that are indicative of data quality. For our analysis, we orientated ourselves around the conceptual framework created by Richard Wang and Diane Strong (11). Their work, often cited in the field of information systems, identifies several key dimensions of data quality, which are critical for ensuring that the information being used or analyzed is reliable, accurate, and useful. By using these categories derived from Wang and Strong’s framework, our evaluation of ChatGPT’s responses not only adheres to established principles of data quality but also provides a structured and thorough method for assessing the effectiveness and reliability of AI-generated information.

We chose five categories to characterize the answers given by ChatGPT:

1. *Ease of understanding*: Was the answer clearly and precisely formulated in a way that was easy to understand?
2. *Concise representation*: Was the answer clearly structured and divided into sections that facilitated readability?
3. *Accuracy*: Did the facts mentioned in the answer correspond to the current scientific literature? Were the statements logical and understandable?
4. *Completeness*: Was the answer complete, and were all aspects of the question adequately addressed? Was important information omitted, or were there unnecessary details?
5. *Relevance*: Was the answer directly related to the question asked, or was there any ambiguity in the answer?

Three medical experts in the field of OB/GYN with long clinical experience individually assessed each answer independently with regard to the five items above using a five-point Likert scale (ranging from 1 = “completely disagree” to 5 = “completely agree”). Mean values from their responses were used for further statistical analysis to ensure consistency of the data.

Subsequently, for every question that had received an incorrect response, we categorized the reason for the error into one of the three options listed below. The authors responsible for conducting the qualitative analysis of the responses (i.e., MR, BM, and FR) collaborated on the analysis and resolved any ambiguous labels.

1. *Incorrect external information.*
2. For the question, ChatGPT used incorrect external information that could not be directly derived from the content of the question.
3. *Failure to consider information within the question.*
4. ChatGPT did not consider information mentioned in the question when generating the answer.
5. *Incorrect linkage of external knowledge with information within the question.*
6. ChatGPT considered the information in the question but combined it incorrectly with correct external knowledge.

ChatGPT’s responses to every question in both datasets were further analyzed for the presence and quantity of “non-obvious insights” as described and applied by Kung et al. (12). In the authors’ recent publication, an insight was defined by a section of the answer that was characterized by the following four items:

1. *Non-definitional*: Did not simply define a term in the input question.
2. *Unique*: A single insight may have been used to eliminate several answer choices.
3. *Non-obvious*: Required deduction or knowledge external to the question.
4. *Valid*: Clinically or numerically accurate; preserved directionality.

Using Kung et al. as an example, we then established an index (“density of insights”) by normalizing the number of insights to the word count for each response generated by ChatGPT (number of insights/word count *100). The significance of the “density of insights” index in our case lies in its ability to quantify the richness and depth of information provided by ChatGPT. In medical contexts, where every word can carry significant weight and the efficiency of communication is crucial, this index helps in assessing whether ChatGPT is providing dense and meaningful content that goes beyond superficial explanations. By focusing on non-obvious insights—those elements of a response that are not immediately apparent, require deeper knowledge or deductive reasoning, and are clinically or numerically accurate—the index ensures that the evaluated content is not only informative but also relevant and applicable in a real-world setting.

### Statistical analysis

The data were evaluated descriptively using Excel (Microsoft®) or Prism (Version 9, GraphPad®). Unpaired chi-square tests were used to determine whether the question difficulty or the type of question (i.e., general knowledge vs. clinical case) had significantly affected ChatGPT’s performance in either dataset. These tests are ideal for comparing categorical variables and are appropriate for assessing whether there are significant differences in the distribution of correct and incorrect answers across these categories. The McNemar statistical test was used to assess whether the availability of multiple-choice answer options had an impact on the accuracy of ChatGPT in answering the questions. The test is used for paired nominal data. Comparing the performance of the same entity (ChatGPT) under two scenarios (with and without multiple-choice options), this test is suitable for evaluating whether these conditions lead to a statistically significant difference in performance. Unpaired *t*-tests were used to compare the word count between correct and incorrect answers generated by ChatGPT, the expert assessment of ChatGPT’s responses via Likert scale ratings and to analyze the density of insight. These tests are used to compare the means of two independent groups. All *p*-values < 0.05 were defined as statistically significant. Tables and figures were generated in Word (Microsoft®) and Prism (Version 9, GraphPad®).

## Results

### ChatGPT delivered comparable and consistent results

The dataset of questions from the OB/GYN course included a total of 160 questions, each of which contained five multiple-choice answer options. On average, the medical students in our survey answered 83.1% (95% CI = 80.0–86.2%) of these questions correctly. Average student results for questions (*n* = 35) from spring semester 2022 were unavailable. In the same dataset, ChatGPT provided correct answers 85.6% (*n* = 137) of the time. In order to test the consistency of ChatGPT’s answers, we conducted a second individual validation round. In this second round, ChatGPT achieved similarly good results for the dataset, with 88.7% (*n* = 142) of answers being correct. Overall, ChatGPT provided consistent results for 91.6% (*n* = 145) of the questions in the validation round compared with in the first round of testing.

The dataset of the medical sate exam included a total of 104 questions. Students answered 73.4% (95% CI = 69.0–77.8%) of these questions correctly. Four questions had to be removed because they included images that ChatGPT could not process. Moreover, two questions were removed because ChatGPT could not commit to one single answer option. Of the remaining 98 questions, ChatGPT answered a total of 70.4% (*n* = 69) correctly.

### ChatGPT maintained its performance without the need for multiple-choice answers

In order to further explore how the answer choices in the exam questions influence the way ChatGPT forms answers and solutions to clinical case presentations, ChatGPT had to solve the questions again, but this time without the five provided multiple-choice answers for each question. Of a total of 46 clinical case presentation questions from the OB/GYN course dataset, five negative questions (“which answer is incorrect?”) had to be excluded because the answers given by ChatGPT could not be assessed without the multiple-choice answer options. ChatGPT’s performance tended to be slightly worse (80.5%; *n* = 33 out of 41) without the multiple-choice answer options in comparison with its performance when the answer choices were provided for the whole group of clinical case presentations (84.8%; *n* = 39 out of 46). However, this difference was not statistically significant (*p*-value = 0.13).

### The difficulty of the questions interfered with ChatGPT’s performance in the OB/GYN course, but not in the state exam questions

We aimed to investigate whether the accuracy of ChatGPT’s answers depended on the difficulty level of the clinical questions. Initially, we categorized questions as *easy* or *difficult* based on their rate of correct answers, which had to be either above or below the mean of correct answers achieved by the medical students across all questions in the datasets (83% for the OB/GYN course dataset and 73% for the state exam dataset). We found that ChatGPT’s performance in the OB/GYN course dataset did not significantly depend on the level of difficulty of the question (*p*-value = 0.1). However, in the medical state exam dataset, ChatGPT’s performance on questions that had been defined as *easy* was significantly better (*p*-value <0.01) (Table 1). Furthermore, we assigned a numerical difficulty score (ranging from 1 = “very easy” to 5 = “very difficult”) to each question in the medical state exam dataset and correlated it with ChatGPT’s performance. We observed a decline in the rate of correct answers from *n* = 27 (27.6%) for Level 1 difficulty to *n* = 1 (1.0%) for Level 5 difficulty. Correspondingly, there was a rise in the rate of incorrect answers from *n* = 2 (2.0%) for Level 1 difficulty to *n* = 8 (8.2%) for Level 5 difficulty (*p*-value <0.001) (Table 2).

**Table 1:** ChatGPT’s performance on questions from the OB/GYN course and the state exam.

		Performance	Question difficulty		
		Overall, *n* (%)	“easy,” *n* (%)	“difficult,” *n* (%)	*p*-value
OB/GYN course	Correct	104 (83.2)	69 (55.2)	35 (28.0)	0.1
	Incorrect	21 (16.8)	10 (8.0)	11 (8.8)	
State exam	Correct	69 (70.4)	51 (52.0)	20 (20.5)	< 0.001
	Incorrect	29 (29.6)	9 (9.1)	18 (18.4)	

The level of difficulty for each question was based on whether it had been answered correctly by more or less than the mean rate of students, which was 83% for the OB/GYN course and 73% for the state exam questions.

**Table 2:** ChatGPT’s performance on the state exam questions.

		Question difficulty, *n* (%)					
		1	2	3	4	5	*p*-value
State exam	Correct	27 (27.6)	22 (22.4)	13 (13.3)	6 (6.1)	1 (1.0)	< 0.001
	Incorrect	2 (2.0)	8 (8.2)	6 (6.1)	5 (5.1)	8 (8.2)	

The level of difficulty for each question is depicted from 1 = “very low” to 5 = “very high,” as assigned by AMBOSS®. For statistical analysis, the numerical difficulty scores 1–3 were depicted as “easy” and 4–5 as “difficult.” Unpaired chi-square test was used to calculate *p*-values.

### ChatGPT answered simple knowledge and patient case questions equally well

For both datasets, we separated questions into one of the following groups: general knowledge questions or clinical case studies. We tested whether the type of question (i.e., knowledge vs. clinical case) altered ChatGPT’s performance. For both the OB/Gyn course (*p*-value = 0.84) and the state exam questions (*p*-value = 0.42), we did not find significant differences between the two types of questions (Table 3).

**Table 3:** Correct and incorrect answers by ChatGPT for each type of question (general knowledge or clinical case presentation).

		Type of question		
		Knowledge, *n* = (%)	Clinical case presentation, *n* = (%)	*p*-value
OB/GYN course	Correct	98 (61.3)	39 (24.3)	0.84
	Incorrect	16 (10.0)	7 (4.4)	
State exam	Correct	30 (30.6)	41 (41.8)	0.42
	Incorrect	9 (9.2)	18 (18.4)	

Unpaired chi-square test was used to calculate *p*-values.

### ChatGPT delivered high-quality answers, especially for questions that were answered correctly

In our qualitative analysis of the reasons for ChatGPT’s incorrect answers in the dataset of the OB/GYN course, the main reason was found to be “incorrect internal knowledge” (*n* = 20; 76.9%), followed by “incorrect connection of internal knowledge with external information in the question” (*n* = 4; 15.4%) and “no consideration of the external information provided in the question” (*n* = 2; 7.7%).

For further qualitative analysis, three medical experts from the field of OB/GYN assessed ChatGPT’s answers using a five-point Likert scale (from 1 = “completely disagree” to 5 = “completely agree”). The experts found a positive assessment for both correct and incorrect answers with regard to the qualities of “ease of understanding” (mean Likert scale = 4.8 for correct answers and 4.6 for incorrect answers) and “concise representation” (mean Likert scale = 4.2 for correct answers and 3.9 for incorrect answers). By contrast, the qualities of “accuracy,” “completeness,” and “relevance” were found to have been significantly better assessed (*p*-value <0.0001) for the correct answers compared with for the incorrect answers (Table 4).

**Table 4:** Mean Likert scale (“1” = completely disagree; “5” = completely agree), with standard deviation (SD) for correctly and incorrectly answered questions by ChatGPT and the mean assessment by three medical experts depicted for each question.

		Ease of understanding	Concise representation	Accuracy	Completeness	Relevance
Incorrect	Mean	4.6	3.9	2.0	3.5	3.6
	SD	0.4	0.8	0.8	1.3	1.3
Correct	Mean	4.8	4.2	4.8	4.6	4.8
	SD	0.3	0.7	0.3	0.4	0.3
*p*-value		0.0268	0.0390	< 0.0001	< 0.0001	< 0.0001

Unpaired *t*-test was used to calculate *p*-values.

### ChatGPT provided a high density of non-obvious insights

**Figure 1:**
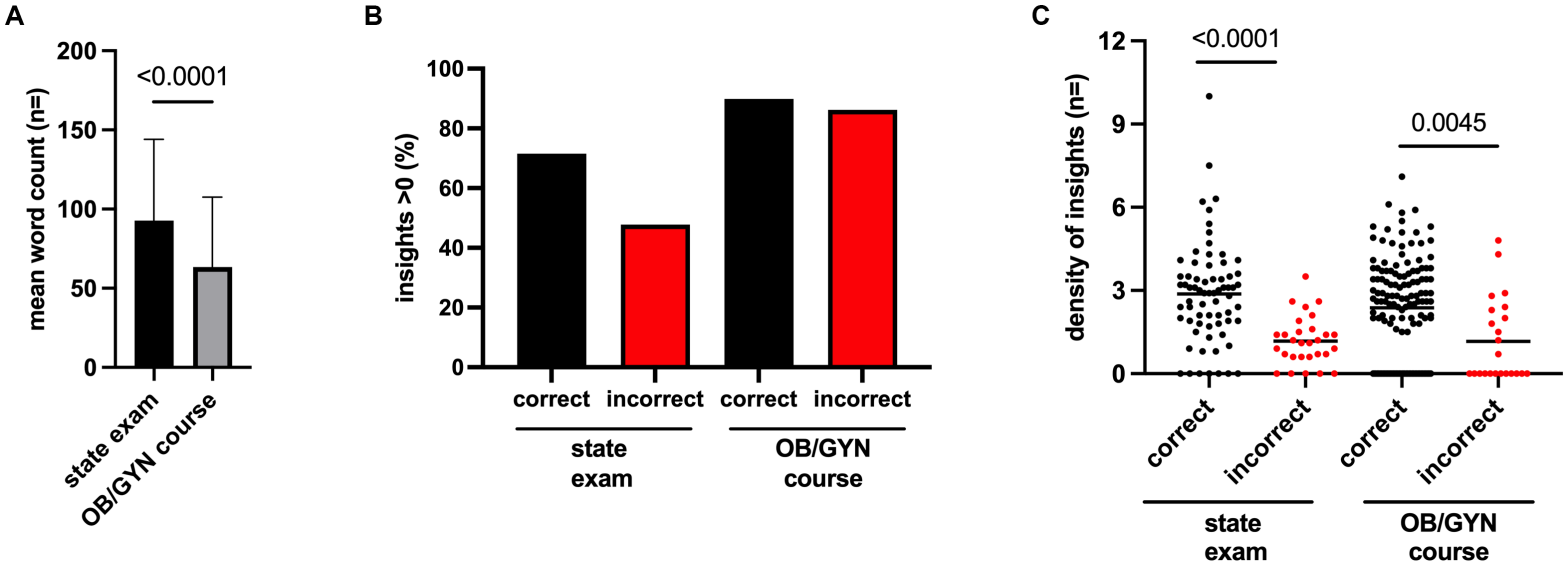
**(A)** Bar diagram depicting the mean word count, with standard deviation for ChatGPT’s answers to the state exam and OB/GYN course questions. **(B)** Bar diagram depicting the percentage with at least a non-obvious insight for questions from the state exam and the OB/GYN course, differentiated by questions answered incorrectly or correctly by ChatGPT. **(C)** Scatter plot depicting the “density of insights” (number of insights/word count *100) of the responses by ChatGPT for the correctly or incorrectly answered questions from the state exam or the OB/GYN course. Each dot represents one answer by ChatGPT; the horizontal line represents mean. Unpaired *t*-test was used to calculate *p*-values. All *p*-values are shown as full digits.

After assessing the quality and consistency of ChatGPT, we continued to evaluate the tool’s potential capacity to enhance autonomous medical learning in the field of OB/GYN. ChatGPT provided answers with a significantly larger mean word count (*p*-value <0.0001) of 93 words for the state exam questions compared with 63 words for the OB/GYN course questions (Figure 1A). We further analyzed the quantity of non-obvious insights provided by ChatGPT. More than 85% of both the correctly and incorrectly answered OB/GYN course questions provided at least one non-obvious insight. With regard to the state exam question, however, the incorrect answers, in particular, demonstrated a decrease in the number of questions with at least one significant insight (Figure 1B). In order to better assess non-obvious insights generated by ChatGPT, we established a density index by normalizing the number of insights to the word count for each response generated by ChatGPT. Thus, we aimed to quantify ChatGPT’s ability to provide knowledge in OB/GYN in a correct, concise, and relevant manner for medical education. In so doing, we noticed that the density of the insight index was significantly higher for questions that had been answered correctly compared with for those that had been answered incorrectly for both the state exam (*p*-value <0.0001) and the OB/GYN course question (*p*-value <0.0045) (Figure 1C).

## Discussion

ChatGPT has gained significant attention from the public and media alike since its release in late 2022 (13–15). The tool surpassed 100 million users in January 2023, making it the fastest-growing consumer application to date (16). ChatGPT has been recognized both for its potential to revolutionize the way in which we interact with machines and for its ability to pave the way for the transformation of entire industries, such as media, marketing, and computer science (17). Some have even argued that ChatGPT may represent the beginning of a new industrial age in which AI and its applications will have a similar impact on economies and societies as the Internet had at the beginning of the 1990s (18). The fact that this new technology could also expand to other fields – such as medical education and clinical practice – is therefore not only possible, but also rather likely (19, 20).

Our study yielded novel findings that highlight the proficiency of ChatGPT to perform intricate tasks related to handling complex medical and clinical information in the field of OB/GYN. ChatGPT provided consistent answers and explanations to medial problems and did not require help in finding correct solutions to medical problems in multiple-choice answer options. Remarkably, ChatGPT demonstrated comparable results with those achieved by medical students in the setting of a real exam after extensive studying and exam preparation. Indeed, referring to the usual exam passing score of 60%, ChatGPT was capable of passing both our OB/GYN course examination (83.1%) and the OB/GYN share of the German medical licensing examination (73.4%). It is important to note that we refrained from providing any prompts or training to the AI during the experiment, and we systematically cleared the AI session before inputting each question variant in order to avoid a chain-of-thought bias (12).

Our results from the OB/GYN-related exams are in line with or slightly better than ChatGPT’s performance in recent publications with regard to questions from the United States Medical Licensing Exam (USMLE) and the National Board of Medical Examiners (NBME) (10, 12). Interestingly, ChatGPT demonstrated particularly good results on questions from the OB/GYN course in our study. We hypothesize that the characteristics of the exam questions were crucial for obtaining such a result. In comparison with the state exam question, the OB/GYN course questions were significantly shorter (mean word count = 23 vs. 102 words), and the quality of the questions was different. The OB/GYN course questions were rather direct and required generally factual knowledge (e.g., “What is NOT a risk factor for the development of ovarian cancer?”), whereas the state exam questions addressed more complex medical questions (e.g., “A 64-year-old woman presents at a gynecological practice with vaginal bleeding that has been ongoing for 14 days. The patient weighs 90 kg and is 150 cm tall. She has been suffering from type 2 diabetes mellitus for 5 years, which is being treated with antidiabetic medication. The patient reports having consistently high blood pressure but is not following an antihypertensive therapy prescription. The suspected diagnosis of an early-stage malignancy (FIGO stage IA) has been confirmed. Which of the therapeutic options listed below is indicated next?”). The questions from the state exam seem to have been more challenging for both the medical students and ChatGPT. This higher level of difficulty of the state exam questions could be explained by the fact that they require both more complex processing and the weighing of multiple pieces of clinical information as compared with the OB/GYN course questions. This finding corresponds to the main reason that we identified for the incorrect answers given by ChatGPT: The primary cause was attributed to the processing of “incorrect internal knowledge,” which accounted for 76.9% of the errors, while a significantly smaller percentage (7.7%) of the errors were attributed to “not considering the external information provided in the question.” ChatGPT’s insufficiencies could have been caused by an inadequately trained model, for example, due to the underrepresentation of clinically relevant and distinctive medical knowledge. Additionally, the human factor may have contributed to this finding because insufficient or invalid human judgment during the initial reinforcement stages could have affected the model’s performance (12).

Our analysis underlines the positive aspects of ChatGPT and its potential “real-world” application in medical education and clinical practice. With its use of machine-learning techniques, ChatGPT demonstrated high adaptability and accuracy in resolving medical problems. By implementing this approach, ChatGPT is able to thoroughly assess the context of a query and generate tailored responses that are customized to individual users (21). Moreover, the introduction of a conversational interface in ChatGPT enhances its usability, and the ability to ask follow-up questions enables users to gain a more comprehensive understanding of the concepts addressed in their queries, thereby enabling the tool to do more than merely output answers. However, it is important to acknowledge that the answers provided by ChatGPT are limited to the data on which the tool was trained and rely on patterns learned from vast amounts of text data. As a consequence, ChatGPT’s responses may not always be up-to-date or entirely accurate for all scenarios (21). In a recent publication, Weng et al. stated that ChatGPT had failed Taiwan’s Family Medicine Board Exam (22). The authors hypothesized that possible factors that had contributed to these results included the challenging nature of the specialist exam in family medicine and the limited availability of comprehensive traditional Chinese-language resources for processing medical problems. Despite these challenges, the authors argued that ChatGPT had demonstrated satisfactory performance in handling negatively phrased questions, mutually exclusive questions, and case-scenario questions, thereby suggesting its potential as a valuable learning and exam-preparation tool (22).

The results of our study indicate that ChatGPT is at the forefront of advancements in the area of machine learning tools and that it displays significant improvements and capabilities in answering medical issues because open-domain-question answering models have faced considerable challenges in solving medical problems until recently. For instance, Jin et al. achieved an accuracy rate of 36.7% on a dataset of 12,723 questions derived from Chinese medical licensing exams in 2020 (23). Similarly, Ha et al. reported only 29% accuracy on 454 USMLE Step 1 and Step 2 questions in 2019 (24). Improved results were seen in the biomedical question answering (QA) dataset collected from PubMed abstracts from 2019 (PubMedQA). This model attained an accuracy rate of 68.1% and was designed to answer only yes-or-no questions by using information sourced from the corpus of PubMed-available abstracts (25). Interestingly, ChatGPT outperformed PubMedGPT, a language model with a similar neural structure but that had been trained solely on biomedical literature and that had achieved an accuracy rate of only 50.3% (26). As argued by Kung et al., this difference in performance may be attributed to PubMedGPT’s domain-specific training, from which it might have absorbed ambiguous information from ongoing academic discourse, thereby leading to less conclusive responses (12).

The landscape of medical problem-solving tools is diverse, and comparing ChatGPT’s capabilities to other commercially available solutions is vital for a comprehensive understanding of its position. Traditional Medical Decision Support Systems (MDSS) like UpToDate (27) and ClinicalKey (28), which rely on curated, evidence-based content, offer structured and highly processed medical information. While these systems are renowned for their accuracy and reliability, they lack the conversational interface and adaptability of AI-driven tools like ChatGPT. On the other hand, emerging AI-driven solutions, such as IBM Watson for Health (29), offer more dynamic interactions and the ability to process natural language queries, but they may still face challenges in areas like context understanding and the latest data incorporation. Unlike these systems, ChatGPT brings a unique blend of conversational ease and a vast database of information, though it may currently not match the specialized, up-to-date medical knowledge of dedicated MDSS.

Despite its great potential, the integration of AI tools like ChatGPT in medicine necessitates careful consideration of several ethical concerns (30). Firstly, in medical decision-making, the risk of over-reliance on AI could compromise the essential human element in healthcare, particularly in nuanced fields like OB/GYN. While AI can augment the decision-making process, it cannot replace the critical judgment and empathetic understanding inherent to medical professionals. Secondly, the accuracy and currency of data in AI systems are pivotal, especially in fast-evolving fields like medicine. AI’s reliance on historical data may lead to outdated or incomplete medical advice, underscoring the need for continual updates and human oversight. Thirdly, data privacy and security are paramount in handling sensitive medical information. AI systems must ensure robust protection against data breaches and adhere to stringent privacy regulations.

The COVID-19 pandemic has shed a light on the lack of digitalization in medical education and training both in Germany and worldwide. New initiatives have focused on moving away from the conventional lecture-based teaching model and instead prioritize self-directed learning methods that cater to the individual needs that students have (31). Additionally, these endeavors incorporate the use of innovative (online) technologies for enhancing overall educational success (32, 33). The experiences during the COVID-19 pandemic have given rise to various experimental teaching concepts. For instance, in a sub-Saharan African setting, instant messaging platforms such as WhatsApp® have been utilized for distance teaching (34). Furthermore, one German university hospital introduced realistic e-learning cases within a symptom-based curriculum for internal medicine (35). Another approach involved the implementation of virtual “serious gaming” as an alternative to intensive small-group teaching (36). In spite of their innovative ideas, however, none of these concepts is likely to be implemented on a broader scale in medical education. This situation highlights an advantage that ChatGPT and similar AI-based applications have because they are freely available over the Internet, which makes them easily accessible for practical use in students’ everyday learning.

As an interactive learning aid, AI can provide an accessible platform for reviewing and dissecting complex clinical cases and patient scenarios that medical students might not yet encounter in their training (37). Simulated conversations and diagnostic exercises are another area where AI can play a pivotal role. Through these simulations, students can practice their diagnostic skills, receive immediate feedback, and learn to navigate patient interactions effectively. These simulations can also be tailored to mimic a wide range of clinical situations, from common ailments to rare diseases, providing a safe and controlled environment for students to work on their clinical reasoning and decision-making skills. Moreover, AI can be integrated into various educational formats, such as virtual classrooms, online courses, and mobile applications, offering flexibility and convenience for students. It also provides an opportunity for continuous learning outside of traditional classroom settings, making medical education more accessible and adaptable to individual learning preferences (31).

ChatGPT shows special potential as a surrogate for small-group learning (10), which has been proven to be a highly effective teaching approach (36, 38). Small-group learning is characterized by three distinct elements: namely active participation, clear and specific tasks, and facilitated reflection by the participants (39, 40). ChatGPT can demonstrate all three of these essential characteristics of small-group learning, thereby making it a viable alternative for small-group education. One of the significant advantages of using ChatGPT in this role is its accessibility and availability. Small-group learning can be challenging to organize due to scheduling conflicts or limited resources, but ChatGPT offers an on-demand and self-paced learning experience. Learners can interact with the AI model at their convenience, thereby enabling flexibility in their learning journey. In our own study from 2022 that investigated the learning experiences of medical students at our faculty during the COVID-19 pandemic, the aspects of time and spatial flexibility were particularly praised and valued by our students (41). Furthermore, ChatGPT can cater to individual learning needs. In small-group settings, the pace and content of discussions might be influenced by the dynamics of the group, thereby leaving some students with unaddressed questions or uncertainties (42). ChatGPT, on the other hand, can provide personalized responses to individual queries, thus ensuring that each student’s specific knowledge gaps are filled. Another advantage of ChatGPT lies in its potential for a more inclusive learning environment. In some small-group settings, students might feel hesitant to participate actively due to various factors, such as shyness or language barriers. As an AI interface, ChatGPT eliminates such barriers and provides a non-judgmental and non-intimidating platform with which learners can engage and ask questions freely. However, it is also important to acknowledge the limitations of ChatGPT as a surrogate for small-group learning. Indeed, ChatGPT lacks the interactivity and dynamic discussions of small-group settings. Learning in small groups allows for collaborative problem-solving, peer-to-peer feedback, and the exchange of diverse perspectives. These interactive elements foster critical thinking and deeper understanding, which ChatGPT cannot fully replicate. There is also a potential risk of undermining critical thinking and problem-solving skills among students. The ease of accessing information from AI could lead to a dependency that detracts from deeper engagement and intellectual development. Balancing these aspects is crucial for the responsible and effective use of AI in these critical sectors.

The fact that the application of AI will extend beyond academic support to direct patient care and clinical decision-making is obvious. As an advanced AI-driven tool, it can assist healthcare providers by offering quick access to medical information, suggesting potential diagnoses, and providing drug information, thereby acting as a supportive tool for decision-making (30). In patient interactions, ChatGPT could be employed for patient education, explaining medical conditions and treatments in easily understandable language, and for gathering preliminary patient histories, thus streamlining the pre-consultation process. In the near future, we can envision AI systems aiding in diagnostic processes by analyzing patient data, symptoms, and medical histories, thereby providing clinicians with potential diagnoses or highlighting overlooked aspects of a patient’s condition. This has been thoroughly discussed in transformative fields like radiology and pathology, where AI’s image recognition capabilities can augment human expertise (43, 44). Moreover, AI can play a crucial role in personalized medicine. By analyzing large datasets, including genetic information, AI can help tailor treatments to individual patients, enhancing the efficacy and reducing the side effects of therapies (45). In patient management, AI tools can assist in monitoring chronic conditions, alerting healthcare providers to changes in a patient’s status, and suggesting adjustments to treatment plans (46). Furthermore, ChatGPT could be integrated into electronic health records (EHRs) systems, assisting with documentation tasks, reducing administrative burdens, and allowing clinicians more time for patient care (47). However, it is crucial that these applications are monitored and guided by healthcare professionals to ensure accuracy and ethical use, particularly in dealing with sensitive patient data and making clinical decisions. The integration of ChatGPT into medicine and clinical care promises not only to enhance efficiency but also to improve the quality of patient education and engagement, ultimately contributing to better healthcare outcomes.

### Limitations

The present study on ChatGPT’s performance in clinical problems entailed several potential limitations that should be considered in the context of medical education and clinical practice. The limited dataset size is a first point of concern. Our reliance on a dataset of OB/GYN course questions and state exams may not have adequately captured the vast range of medical knowledge and question types that are encountered in real-world scenarios. Therefore, the use of a more extensive and diverse dataset would have provided a more comprehensive evaluation of ChatGPT’s abilities. Moreover, the fact that the study focused solely on course questions and state exams in the field of OB/GYN may call into question the generalizability of our data. Each medical specialty has its unique complexities, terminologies, and practice nuances, which may not be uniformly comprehensible or addressable by a generalized AI tool like ChatGPT. For instance, the diagnostic reasoning in psychiatry, in surgical fields, or in emergency medicine present different challenges that may not be fully captured in the OB/GYN examination context. However, recent research in medical education and the potential application of ChatGPT in diverse educational and clinical settings has shown that ChatGPT shows general applicability and could serve as a valuable tool for medical educators, students, and clinicians alike (6, 48–51). In fact, a fundamental advantage of AI is its high flexibility, allowing it to be applied effectively in various settings without diminishing its impact.

The study’s results regarding the impact of question difficulty on ChatGPT’s performance may also raise concerns. While ChatGPT demonstrated consistent performance in the OB/GYN course dataset, its effectiveness was influenced by the complexity of the questions in the state exam. This finding raises doubts about ChatGPT’s adaptability to varying levels of difficulty and emphasizes the need both for further fine-tuning and for incorporating data that could enhance the tool’s performance across all contexts. Another potential limitation is the inability of ChatGPT to process images. In medical education, visual information – such as radiological images and anatomical diagrams – plays a vital role. The fact that ChatGPT struggles with image processing currently restricts its applicability in real-world medical scenarios in which such visual content is very commonly encountered. However, the tool’s newest version – ChatGPT-4 – addresses this shortcoming by implementing image-analysis features that are able to identify objects in photos (52). This innovation may indeed only mark the beginning of further advancements in this area.

All of these limitations are, however, temporary. In the next decade, ChatGPT is expected to undergo transformative advancements, primarily driven by breakthroughs in artificial intelligence, machine learning, and natural language processing. We can anticipate a more nuanced understanding of language and context, including better handling of cultural nuances and idioms, achieved through sophisticated algorithms and diverse, expansive training datasets. Enhanced contextual awareness will make interactions more coherent over longer conversations, while personalization features will tailor responses to individual user preferences and histories. Multimodal capabilities will likely be integrated, allowing ChatGPT to process and respond to text, voice, and visual inputs. Real-time learning from interactions will continuously update its knowledge base, and efforts have to be made to ensure ethical use and bias mitigation.

## Conclusion

Our data provide valuable insights into ChatGPT’s role in medical education and clinical practice, particularly in the field of OB/GYN. The findings suggest that ChatGPT demonstrates promising potential as a supplementary tool for medical students and healthcare professionals alike. ChatGPT’s ability to provide accurate and insightful responses to medical questions showcases its adaptability to complex clinical scenarios. The study’s insights into ChatGPT’s capabilities within OB/GYN are particularly significant, hinting at broader implications for its application across various medical settings. As AI technology continues to advance, it is necessary to continue evaluating and refining tools like ChatGPT to ensure they meet the evolving needs of medical education and practice, while also addressing ethical considerations and maintaining the highest standards of patient care.

## Data availability statement

The raw data supporting the conclusions of this article will be made available by the authors, without undue reservation.

## Ethics statement

The present study was conducted in accordance with the Declaration of Helsinki (2008 version). Since the data stem from anonymously analyzed exam questions, no further consultation or approval by the ethics committee was required, as is outlined in the online guidelines by the Joint Ethics Committee of the Universities of Bavaria (https://www.gehba.de/fileadmin/daten/Gehba/GEHBa-FAQ_2.1.pdf).

## Author contributions

MR: Conceptualization, Data curation, Formal analysis, Visualization, Writing – original draft, Methodology, Project administration. KK: Data curation, Investigation, Writing – review & editing. AS: Data curation, Investigation, Writing – review & editing. VR: Data curation, Investigation, Writing – review & editing. NA: Data curation, Investigation, Writing – review & editing. AG: Data curation, Investigation, Writing – review & editing. FlR: Supervision, Writing – review & editing. EK: Resources, Supervision, Writing – review & editing. MK: Resources, Supervision, Writing – review & editing. FaR: Conceptualization, Writing – original draft. BM: Conceptualization, Data curation, Formal analysis, Visualization, Writing – original draft.
